# Ankle motion influences the external knee adduction moment and may predict who will respond to lateral wedge insoles?: an ancillary analysis from the SILK trial

**DOI:** 10.1016/j.joca.2015.02.164

**Published:** 2015-08

**Authors:** G.J. Chapman, M.J. Parkes, L. Forsythe, D.T. Felson, R.K. Jones

**Affiliations:** † School of Health Sciences, University of Salford, Salford, UK; ‡ Arthritis Research UK Epidemiology Unit, Centre for Musculoskeletal Research, University of, Manchester, Manchester, UK; § NIHR Manchester Musculoskeletal Biomedical Research Unit (BRU), Manchester Academic Health Sciences Centre, Manchester, UK; ‖ Clinical Epidemiology Unit, Boston University School of Medicine, Boston, MA, USA

**Keywords:** Osteoarthritis, Knee, Lateral wedge insoles, Biomechanical response, External knee adduction moment

## Abstract

**Objective:**

Lateral wedge insoles are a potential simple treatment for medial knee osteoarthritis (OA) patients by reducing the external knee adduction moment (EKAM). However in some patients, an increase in their EKAM is seen. Understanding the role of the ankle joint complex in the response to lateral wedge insoles is critical in understanding and potentially identifying why some patients respond differently to lateral wedge insoles.

**Method:**

Participants with medial tibiofemoral OA underwent gait analysis whilst walking in a control shoe and a lateral wedge insole. We evaluated if dynamic ankle joint complex coronal plane biomechanical measures could explain and identify those participants that increased (biomechanical non-responder) or decreased (biomechanical responder) EKAM under lateral wedge conditions compared to the control shoe.

**Results:**

Of the 70 participants studied (43 male), 33% increased their EKAM and 67% decreased their EKAM. Overall, lateral wedge insoles shifted the centre of foot pressure laterally, increased eversion of the ankle/subtalar joint complex (STJ) and the eversion moment compared to the control condition. Ankle angle at peak EKAM and peak eversion ankle/STJ complex angle in the control condition predicted if individuals were likely to decrease EKAM under lateral wedge conditions.

**Conclusions:**

Coronal plane ankle/STJ complex biomechanical measures play a key role in reducing EKAM when wearing lateral wedge insoles. These findings may assist in the identification of those individuals that could benefit more from wearing lateral wedge insoles.

## Introduction

Knee osteoarthritis (OA) affects approximately 12.5% adults over the age of 65 years old^1–3^. This progressive condition is characterised by pain and stiffness in the joint resulting in difficulty in weight bearing activities including walking and stair climbing^1,2,4^, resulting in adverse effects on loss of function, personal independence and ultimately a reduction in quality of life.

Knee OA commonly affects the medial tibiofemoral compartment of the joint presumably due to the high percentage (up to 60–80%) of the load being transmitted to this compartment of the knee compared to the lateral side^5–8^. During walking the ground reaction force (GRF) passes medial to the knee, creating an external knee adduction moment (EKAM) acting on the knee which adducts the tibia with respect to the femur. The EKAM is widely accepted as a surrogate measure of medial knee loading. The EKAM has been directly linked with lower limb malalignment, disease severity^9^, disease progression^10^ and self-reported pain^11,12^. As focal mechanical loads play an important role in disease severity and progression, reduction in the EKAM can be used as an objective goal for load-modifying conservative treatments designed to reduce load, pain and potentially slow disease progression and thus improve physical function. One common conservative treatment for medial knee OA is the use of lateral wedge insoles which are placed inside patient's shoes in an attempt to redistribute the load on the knees and thus decrease the EKAM. Past research has demonstrated that lateral wedge insoles decrease EKAM in patients with medial knee OA^13–22^.

Individuals' EKAM response to lateral wedge insoles is remarkably variable, with up to 30% of treated patients demonstrating an increase (worsening) in EKAM^15–22^. Randomised controlled trials examining the effects of lateral wedge insoles on knee pain have failed to show significant effects on pain reduction^13,14,23–25^ with a recent meta-analysis showing no significant change in pain when compared to neutral inserts^26^. The modest effect of EKAM reduction and the disappointing effects of pain reduction could be due to the variability in EKAM response to wearing lateral wedge insoles and/or that previous studies have grouped participants together, on the assumption that lateral wedge insoles uniformly reduce EKAM and thereby alleviate knee pain.

The majority of past research examining the effects of lateral wedge insoles on lower limb joints has concentrated on the knee joint. In one study, lateral wedge insoles had little effect on the hip^27^. At the foot, lateral wedge insoles shift the centre of force pressure (COFP) laterally, increasing the ankle eversion moment and shortening the knee-GRF lever arm, thus reducing the EKAM^14,17,20,21,27,28^. These findings suggest that the ankle/subtalar joint complex (STJ) may play a key role in the mechanical mechanism(s) of lateral wedge insoles and medial knee loading. To our knowledge, no previous research has investigated to what extent lateral wedge insoles alter coronal plane foot and ankle biomechanics and/or determine if changes in foot/ankle biomechanics are linked to changes in EKAM in a large cohort of medial knee OA patients. An aim of the present study was to conduct an analysis of ancillary measurements obtained as part of the SILK trial to gain greater insight into the effect of lateral wedge insoles on foot and ankle biomechanics and also to understand the relationship between changes in foot and ankle biomechanics and change in EKAM. Furthermore, given the variability of biomechanical response to lateral wedge insoles, we also examined whether the effects of the lateral wedge insole on foot and ankle biomechanics would identify individuals with reductions in EKAM.

Overall, we hypothesised that by classifying participants by EKAM response, dynamic coronal plane ankle/STJ complex biomechanics can identify and help explain why some patients experience an increase, whereas others show a decrease in EKAM.

## Participants and methods

### Participants

Participants with knee pain were recruited from orthopaedic clinics, physiotherapy clinics, and through advertisements in the local media. Participants were included in the study if they reported at least mild knee pain during walking on a flat surface in the last week, assessed by the Knee Injury and OA Outcome Score pain subscale question P5^29^ (we required that a potential participant scored their pain as either mild, moderate, severe or extreme), were aged between 40 and 85 years old and demonstrated medial tibiofemoral OA Kellgren Lawrence (KL, grade II or III) on radiograph of the affected knee with greater medial than lateral joint space narrowing determined by an experienced academically-based musculoskeletal radiologist. Exclusion criteria included patients experiencing more pain localised in the patellofemoral joint on examination than the medial joint, had tricompartmental knee OA or KL grade 1 or 4 tibiofemoral OA, the latter based on reports that those with KL 4 have not responded to lateral wedge insoles. Other exclusion criteria included any lower limb realignment surgery; total knee replacement of the affected side; any foot or ankle problems that might negate the use of footwear modifying interventions; the use of a walking aid; knee surgery or injections in the previous 6 months or the use of foot orthotics in the past 6 months. The symptomatic knee was the only knee tested in this study. For those patients that had bilateral medial knee OA, the more painful knee was deemed the affected side. NHS Research Ethics approval was obtained for the study and all participants provided written informed consent.

### Interventions

**Fig. 1:**
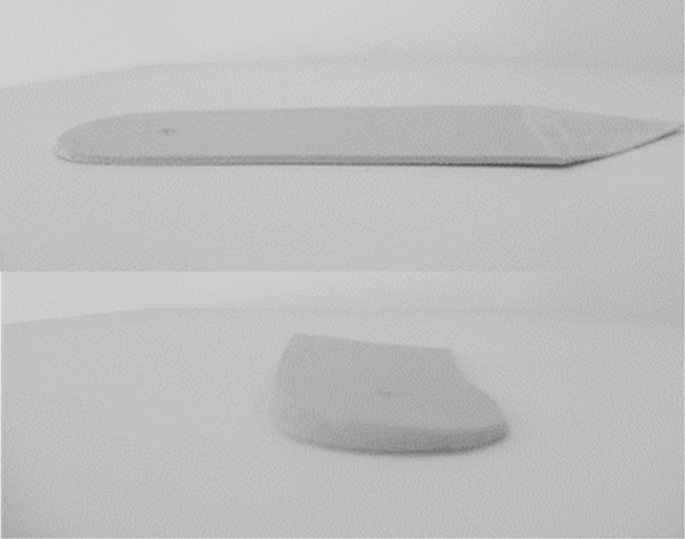
Diagram illustrating the typical lateral wedge insole used in the present study.

As an ancillary study to the SILK trial (ISRCTN: 83706683) which was a single visit randomised trial testing different lateral wedge insoles and shoes for their effect on the EKAM we performed an analysis on foot and ankle motion and how they related to knee moments in one of the conditions, a typical lateral wedge insole. This insole has previously been shown to reduce EKAM in medial knee OA patients^22,30^. The ‘typical’ lateral wedge was comprised of ethylene-vinyl acetate with a Shore A density of 60. This 5° lateral wedge insole post from the heel to the fifth metatarsal head and did not have a medial arch support (see Fig. 1). The control shoe comprised of a flat, thin soled, leather shoe (Ecco Zen, UK). The lateral wedge was inserted into the control shoe bilaterally, with each participant having a five minute familiarisation period for each experimental condition. The order of control vs lateral wedge insole was randomised using computer-generated permutations, concealed in pre-sealed sequentially-numbered envelopes that were generated by the trial statistician, prior to participants' enrolment in the study, who was not present during recruitment or testing.

### Protocol

While wearing each condition, participants underwent 3D gait analysis. A 16 camera Qualisys (Qualisys, Sweden) OQUS3 motion analysis system (collected at 100 Hz) and four force plates (AMTI, USA) (collected at 200 Hz) embedded flush in the ground were used to measure lower limb kinematics and kinetics. Each participant completed a minimum of three successful trials sequentially under each condition, at a self-selected walking speed. A successful trial was defined as a trial in which the participant walked naturally landing the whole foot of the affected limb on the force plate. Participants were not informed about the force plates to minimise the participant from ‘targeting’ the force plates. The CAST marker set technique^31^ was employed whereby rigid clusters of four non-orthogonal markers were positioned over the lateral shank, lateral thigh and sacrum to track the segmental kinematics in six degrees of freedom. Four retroreflective markers (positioned over the first and fifth metatarsal heads, the most posterior aspect of the calcaneus and the most anterior tip of the shoe) were glued securely to the control shoes with the foot being modelled as a rigid, single segment as reported previously^28^. A static calibration trial was collected, for each experimental condition, in which retroreflective markers were placed on bony landmarks to specify the location of the lower limb joints in relation the clusters and to approximate joint centres. Ankle and knee joint centres were calculated as midpoints between the malleoli and femoral epicondyles, respectively. The hip joint centre was calculated using the regression model based on the anterior and posterior superior iliac spine markers^32^. In Visual 3D (C-Motion, Rockville, Maryland), joint kinematics were calculated using an X–Y–Z Euler rotation sequence equivalent to the joint coordinate system^33^ and joint kinetic data were calculated using three-dimensional inverse dynamics. External knee adduction moments were normalised to participant's mass (Nm/kg) and knee adduction angular impulse (KAAI) normalised to participant's mass and time (Nm/kg*s). Additionally, coronal plane biomechanical measures relating to the ankle angle, external ankle eversion moments and the position of the COFP with respect to the foot were also calculated. Centre of force pressure measures were derived from the known location of the shoe (from the markers placed on the foot/ankle) on the force plate. Medio-lateral COFP was defined as the distance of the centre of force with respect to the midline of the foot (vector constructed by the heel and toe markers). A custom Matlab (Matlab, USA) programme was used to extract the peak EKAM during early stance (between 17% and 50% of stance^34^) and to calculate the KAAI. For each additional parameter, data were extracted at the instant of peak EKAM in early stance and the peak value between heel strike and peak EKAM in early stance as this is where medial loading is greatest.

### Data analysis and statistical analysis

In the first part of the analysis, we tested for effects of the lateral wedge insole on coronal ankle/STJ complex variables. Paired *t*-tests were used to examine if each variable of interest changed during the lateral wedge condition, in comparison to the control condition.

The second analysis investigated whether the change in coronal plane biomechanical measures was associated with the change in peak EKAM, when using the lateral wedge. We ran several univariate linear regression models, with the predictor variable for each model being the difference in one of the coronal plane variables of interest, when wearing the lateral wedge. The outcome variable in each model was the difference in EKAM between the lateral wedge and the control condition.

The third part of the analysis categorised participants based on whether their peak EKAM decreased or increased when wearing the lateral wedge insole. A participant with a reduction in peak EKAM in the *lateral* wedge condition was defined as a ‘biomechanical responder’, those with no change or an increase in peak EKAM were ‘biomechanical non-responders’. We then used logistic regression to see which coronal ankle/STJ complex variable could predict response to EKAM (using this dichotomous classification). For this analysis, we used coronal ankle/STJ complex variables from the *control condition only*, as this would test if we could effectively establish which (if any) coronal plane ankle/STJ complex variables were indicative of a response to lateral wedges, prior to an actual test of treatment.

Statistical analysis was performed using Stata version 13.1 (StataCorp, College Station, Texas, US) with the significance level set at *P* < 0.05. Model residuals were checked for normality using residual-versus-fitted plots. Due to the variables of interest all being highly correlated with one another (i.e., highly collinear), all regression models tested (both linear and logistic) considered only one ankle outcome of interest, at any time. Models using several ankle outcomes as predictors simultaneously were avoided, since the highly collinear predictors would cause inappropriately large standard errors.

## Results

### Study sample characteristics

Seventy participants (43 male, 27 female) had medial knee OA (mean age 60.3 years (SD 9.6 yrs), height 1.69 m (0.09), weight 87.3 kg (18.5), BMI 30.5 kg/m^2^ (4.9)). Of the 62 participants with KL data, the mean KL score was 2.63 and ranged from grade 2 to grade 3. We reviewed recent knee arthroscopy reports or MRIs for eight participants who did not have x-rays prior to the study to ensure that these participants also had medial>lateral cartilage loss and other features of OA. There was no difference in walking speed between conditions (mean gait speeds: control condition 1.163 m/s; typical wedge 1.166 m/s).

Table I outlines the difference in coronal plane biomechanical variables when wearing the lateral wedge insole, compared to the control shoe. Notably, the lateral wedge produced an immediate, significant decrease in peak EKAM (overall change from control = −0.023 Nm/kg; 95% CI −0.035 Nm/kg to −0.011 Nm/kg) and KAAI (overall change from control = −0.012 Nm/kg*s; 95% CI −0.016 Nm/kg*s to −0.009 Nm/kg*s). Expressed as a percentage change from the control shoe, these changes reflect a reduction in peak EKAM of 5.85%, and a reduction in KAAI of 7.95%. Please note that the study sample demographics, peak EKAM and KAAI have been reported previously^35^.

Participants' response to wearing the lateral wedge insole varied considerably with 33% (*n* = 23) of participants demonstrating *increased* peak EKAM when wearing the lateral wedge insole (compared to the control shoe). The mean change in peak EKAM for these individuals was an increase of 0.028 Nm/kg (95% CI 0.018 Nm/kg to 0.037 Nm/kg), reflecting an increase of 8.15% compared to the control shoe. The remaining 67% of participants (*n* = 47) showed a decrease in peak EKAM when wearing the lateral wedge insole, compared to the control shoe (mean peak EKAM change −0.048 Nm/kg; 95% CI −0.059 to −0.037 Nm/kg, reflecting a reduction of −11.39% from the control shoe, in this subgroup).

The lateral wedge insole caused the ankle/STJ complex to be in a significantly more everted position (as shown by significantly greater peak ankle angle and ankle angle at peak EKAM) compared to the control condition (see Table I). Overall, the lateral wedge insole caused a significant lateral shift in COFP at peak EKAM and a significantly greater peak COFP compared to the control shoe. Ankle eversion moment at peak EKAM and peak ankle eversion moment were significantly greater under the lateral wedge condition compared to control conditions.

Linear regression analysis revealed no significant relationships between change in coronal plane biomechanical variables under the lateral wedge condition and change in peak EKAM (see Table II).

The final part of the analysis revealed participants with a higher peak ankle eversion angle (OR 1.248; 95% CI 1.025 to 1.519; *P* = 0.027) or a higher ankle angle at peak EKAM (OR 1.241; 95% CI 1.021 to 1.509; *P* = 0.030) in the control condition, were more likely to classified as a biomechanical responder (i.e., EKAM more likely to decrease) to the lateral wedge insole (see Table III), although no clear-cut threshold in ankle angle in the control condition between groups was found (see Supplementary Fig. 1). In additional analyses, we found no difference in findings by gender.

## Discussion

The present paper focuses on dynamic ankle/STJ complex biomechanics and investigated whether ankle biomechanics can assist in understanding and identifying why some individuals benefit (i.e., reduce peak EKAM) when wearing lateral wedge insoles and others do not. Our findings suggest that coronal plane ankle/STJ complex biomechanical parameters play an important role in reducing peak EKAM and may predict those that are likely to experience a reduced peak EKAM when wearing lateral wedge insoles.

In the present study, those patients whose peak EKAM decreased in the lateral wedge condition compared to control (biomechanical responders) made up the majority the sample (67%) and experienced a decrease in their peak EKAM of 11.39% under the lateral wedge condition compared to control. In contrast, the remaining third of participants whose peak EKAM increased in the lateral wedge condition (the biomechanical non-responders), increased their peak EKAM by a mean of 8.15% compared to the control condition. This variability in peak EKAM response to lateral wedge insoles is consistent with past research^15–22^ and may be a factor why reported reductions in peak EKAM are modest^15,17–19,21,22,27,30,36^. Similar reductions in peak EKAM and variability in response have been shown when wearing specialised footwear^37–41^. It is important to highlight that footwear may play a key role in peak EKAM response to interventions. However, more research is required to fully understand this issue. These findings on peak EKAM suggest that grouping participants by peak EKAM response may demonstrate the true biomechanical effects of lateral wedge insoles on knee loading and be able to examine the clinical effects associated with this grouping. However, future research is required to confirm whether grouping participants by coronal ankle biomechanical variables assists in identifying those likely to benefit from wearing lateral wedge insoles and determine if there are clinical measurements that could be utilised instead of expensive 3D gait analysis.

The overall effect of the lateral wedge insoles resulted in a lateral shift in COFP, a more everted ankle/STJ complex and a greater eversion moment compared to the control condition which is consistent with those reported previously^17,20,21,27^. The increased eversion moment and everted ankle/STJ complex as well as the increased laterally displaced COFP suggests that these are potential key biomechanical responses to wearing lateral wedge insoles for reducing medial knee loading especially considering that Hinman *et al.*^17^ showed that shifting the COFP laterally caused the GRF to move towards the centre of the knee, reducing the knee-GRF distance and EKAM. However, the present changes in coronal plane biomechanical variables did not significantly correlate with change in peak EKAM (see Table II), potentially due to the inclusion of participants that had an increase in the EKAM. Therefore, more research is required to determine the mechanism of action of lateral wedge insoles in individuals who have a positive EKAM response.

Predicting and/or identifying if an individual is likely to benefit from an intervention is an important issue. In the present study we attempted to determine if it is possible to predict those individuals that are likely to respond to lateral wedge insoles (i.e., decrease their peak EKAM; biomechanical responders) compared to those that will not respond (biomechanical non-responders), by using coronal plane ankle/STJ complex biomechanical variables within the control condition. Our findings demonstrated that those individuals that have a greater everted ankle/STJ complex under the control condition were more likely to decrease their peak EKAM under lateral wedge conditions (Table III). This finding may be counter-intuitive given that those individuals that have a more inverted foot could potentially have greater range to rotate their foot into eversion. However, the present findings suggest that those patients with a more everted foot are more likely to be shifting the centre of foot pressure more laterally, thus potentially reducing their EKAM compared to those with a more inverted foot, a finding that is consistent with other past biomechanical studies examining foot posture and knee loading^42,43^. Taken together, these findings suggest that those patients with a less everted ankle joint complex under control condition may have restricted frontal plane ankle range of motion, thus when a lateral wedge is inserted into their shoe, the restricted ankle range of motion may not allow the ankle joint complex to evert/pronate sufficiently to alter the load at the knee. However, due to the omission of clinical data, this is only a hypothesis and an area that future research needs to focus on. To our knowledge, this is the first study to demonstrate that it is possible to distinguish if an individual is likely to decrease their peak EKAM when wearing lateral wedge insoles. While this is an important finding, at present, sophisticated 3D motion analysis systems are required to highlight the small coronal plane kinematic differences and this may not be available in all hospitals and/or clinics. Therefore, future research needs to consider the use of a clinical assessment to determine the range of motion of lower limb joints and/or consider foot type to link the clinical assessment with the biomechanical analysis to determine if it is possible to simplify the identification of individuals that are likely to respond to wearing lateral wedge insoles. From a clinical point of view, our regression analyses has some potentially important implications, in that, being able to identify if someone is likely to respond to wearing lateral wedge insoles, the more beneficial the treatment is likely to be for those individuals. Conversely, categorising if someone is unlikely to respond to wearing lateral wedge insoles, other treatments should be prescribed which should be more beneficial (compared to lateral wedge insoles) for those patients. However, whilst these results are encouraging, future research is required to replicate these finding. There are limitations within the present study. Despite having a large sample of participants (*n* = 70), our decision to categorise participants based on biomechanical response to lateral wedge insoles resulted in the sample size per group decreasing and therefore a potential reason for a number of close but insignificant findings. As the prediction of whether an individual is likely to respond to wearing lateral wedge insoles is based on ankle/STJ complex kinematics under the control condition, the type of footwear that was used in the study needs to be taken into account. It is plausible that different footwear characteristics/participants' own shoes may have differing effects on lower limb kinematics and kinetics. Therefore, future research is required to determine how footwear influences biomechanical response to wearing lateral wedge insoles and may also consider employing a more sophisticated foot model that allows for separation between hindfoot (i.e., four markers placed on the heel of the shoe) and forefoot kinematics during shod gait conditions. Another potential limitation of the current work is that we only measured the immediate effect of lateral wedge insoles on peak EKAM response and dichotomised participants based on their immediate peak EKAM response. It is plausible that peak EKAM response to lateral wedge insoles of some participants may have changed if worn longer. Past research has suggested that when specialised footwear is worn for six months, participants have reduced peak EKAM even when not wearing the prescribed specialised footwear^41,44^, suggesting that knee OA patients show neuromuscular adaptation to potential treatments for medial knee OA. Therefore, future research is required to understand if participants adapt to wearing lateral wedge insoles over time.

## Conclusions

In conclusion, we have demonstrated that the ankle/STJ complex plays an important role in the reduction of peak EKAM when wearing lateral wedge insoles. Furthermore, our findings also demonstrate that coronal plane ankle/STJ complex biomechanical measures under the control condition correlate with the likelihood of experiencing a reduction in peak EKAM when wearing lateral wedge insoles. These findings may provide future insights into determining who will respond to lateral wedge insoles.

## Author contributions

DTF and RKJ conceived of the study idea and designed the study. GJC and LF collected and processed the data. GJC, MJP, DTF and RKJ analysed and interpreted the data. GJC drafted the manuscript. All authors revised the manuscript for intellectual content and approved of the final article prior to submission. RKJ (r.k.jones@salford.ac.uk) and DTF (dfelson@bu.edu) take full responsibility for the integrity of the work as a whole, from the inception to the finished article.

## Role of the funding source

Research in Osteoarthritis Manchester (ROAM) is supported by Arthritis Research UK special strategic award 18676. The Arthritis Research UK Centre of Excellence in Epidemiology is supported by grant number 20380. This study is an ancillary study carried out during the SILK trial (ISRCTN: 83706683). This report includes independent research supported by the National Institute for Health Research Biomedical Research Unit Funding Scheme. The views expressed in this publication are those of the author(s) and not necessarily those of the NHS, the National Institute for Health Research or the Department of Health. The funding source had no role in the study design, collection, analysis and interpretation of the data; in the writing of the manuscript; or in the decision to submit the manuscript for publication.

## Competing interests

RKJ may receive royalties from the lateral wedge insoles.

## Figures and Tables

**Table I:** Effect of the typical lateral wedge insole on coronal plane biomechanical variables compared to the control shoe (*N* = 70). NB: the data on EKAM and KAAI has previously been reported^35^ but is also indicated here for clarity

Variable	Control shoe, mean (SD)	Lateral wedge, mean (SD)	Difference, mean (95% CI), *P*
EKAM (Nm/kg)	0.394 (0.160)	0.371 (0.151)	−0.023 (−0.035 to −0.011), <0.001
KAAI (Nm/kg*s)	0.156 (0.071)	0.144 (0.068)	−0.012 (−0.016 to −0.009), <0.001
Ankle angle at peak EKAM (°)	3.457 (2.777)	4.399 (2.844)	0.942 (0.539 to 1.345), <0.001
Peak eversion ankle angle (°)	3.506 (2.770)	4.425 (2.843)	0.919 (0.518 to 1.321), <0.001
Centre of foot pressure at EKAM (mm)^a^	−0.011 (0.006)	−0.015 (0.006)	−0.004 (−0.005 to −0.003), <0.001
Peak centre of foot pressure (mm)^a^	−0.008 (0.007)	−0.013 (0.006)	−0.005 (−0.006 to −0.004), <0.001
Ankle eversion moment at EKAM (Nm/kg)	−0.077 (0.064)	−0.119 (0.062)	−0.043 (−0.050 to −0.035), <0.001
Peak eversion ankle moment (Nm/kg)	−0.091 (0.055)	−0.126 (0.059)	−0.035 (−0.041 to −0.029), <0.001

a Negative values indicate a lateral shift.

**Table II:** Relationship between change in variables of interest and change in peak EKAM when using the typical lateral wedge insole (*N* = 70)

Variable	Association with change during typical lateral wedge use	
	b (95% CI)	*P*
Ankle angle at peak EKAM (°)	0.002 (0.494 to −0.005)	0.494
Peak eversion ankle angle (°)	0.002 (0.509 to −0.005)	0.509
Centre of foot pressure at EKAM (mm)^a^	−0.498 (0.754 to −3.647)	0.754
Peak centre of foot pressure (mm)^a^	0.448 (0.737 to −2.206)	0.737
Ankle eversion moment at EKAM (Nm/kg)	0.061 (0.749 to −0.318)	0.749
Peak eversion ankle moment (Nm/kg)	0.055 (0.808 to −0.394)	0.808

a Negative values indicate a lateral shift.

**Table III:** The association of gait parameters in the control condition with biomechanical response to the typical lateral wedge (*N* = 70)

Variable	OR (95% CI)	*P*
Ankle angle at peak EKAM (°)	1.241 (1.021 to 1.509)	0.030
Peak eversion ankle angle (°)	1.248 (1.025 to 1.519)	0.027
Centre of foot pressure at EKAM (mm)^a∗^	1.047 (0.439 to 2.494)	0.918
Peak centre of foot pressure (mm)^a∗^	1.031 (0.511 to 2.082)	0.931
Ankle eversion moment at EKAM (Nm/kg)^∗^	0.996 (0.921 to 1.077)	0.922
Peak eversion ankle moment (Nm/kg)^∗^	0.980 (0.893 to 1.075)	0.663

^a^Negative values indicate a lateral shift. For outcomes marked (^∗^), odds ratios have been rescaled to reflect a change of 0.01 units, rather than 1 unit.
