# Corrigendum to “Ankle motion influences the external knee adduction moment and may predict who will respond to lateral wedge insoles?: An ancillary analysis from the SILK trial” [Osteoarthr Cartil 23 (2015) 1316–1322]

**DOI:** 10.1016/j.joca.2016.12.005

**Published:** 2017-04

**Authors:** G.J. Chapman, M.J. Parkes, L. Forsythe, D.T. Felson, R.K. Jones

**Affiliations:** † School of Health Sciences, University of Salford, Salford, UK; ‡ Arthritis Research UK Epidemiology Unit, Centre for Musculoskeletal Research, University of Manchester, Manchester, UK; § NIHR Manchester Musculoskeletal Biomedical Research Unit (BRU), Central Manchester University Hospitals NHS Foundation Trust, Manchester Academic Health Science Centre, Manchester, UK; ‖ Clinical Epidemiology Unit, Boston University School of Medicine, Boston, MA, USA

We have been notified by the authors that there was an error in the affiliation of D.T Felsons address (referenced ‘§’) of the above article. The correct address is shown above.

The authors would like to apologise for any inconvenience caused.

