# Supplementary material for: Ankle motion influences the external knee adduction moment and may predict who will respond to lateral wedge insoles?: an ancillary analysis from the SILK trial
Source: Osteoarthritis Cartilage. 2015 Aug;23(8):1316–22. doi: 10.1016/j.joca.2015.02.164 (PMC4523688; doi:10.1016/j.joca.2015.02.164)

**Supplementary Figure 1. Scatterplot illustrating peak ankle angle at EKAM in control condition dichotomised by biomechanical response to typical lateral wedge.**


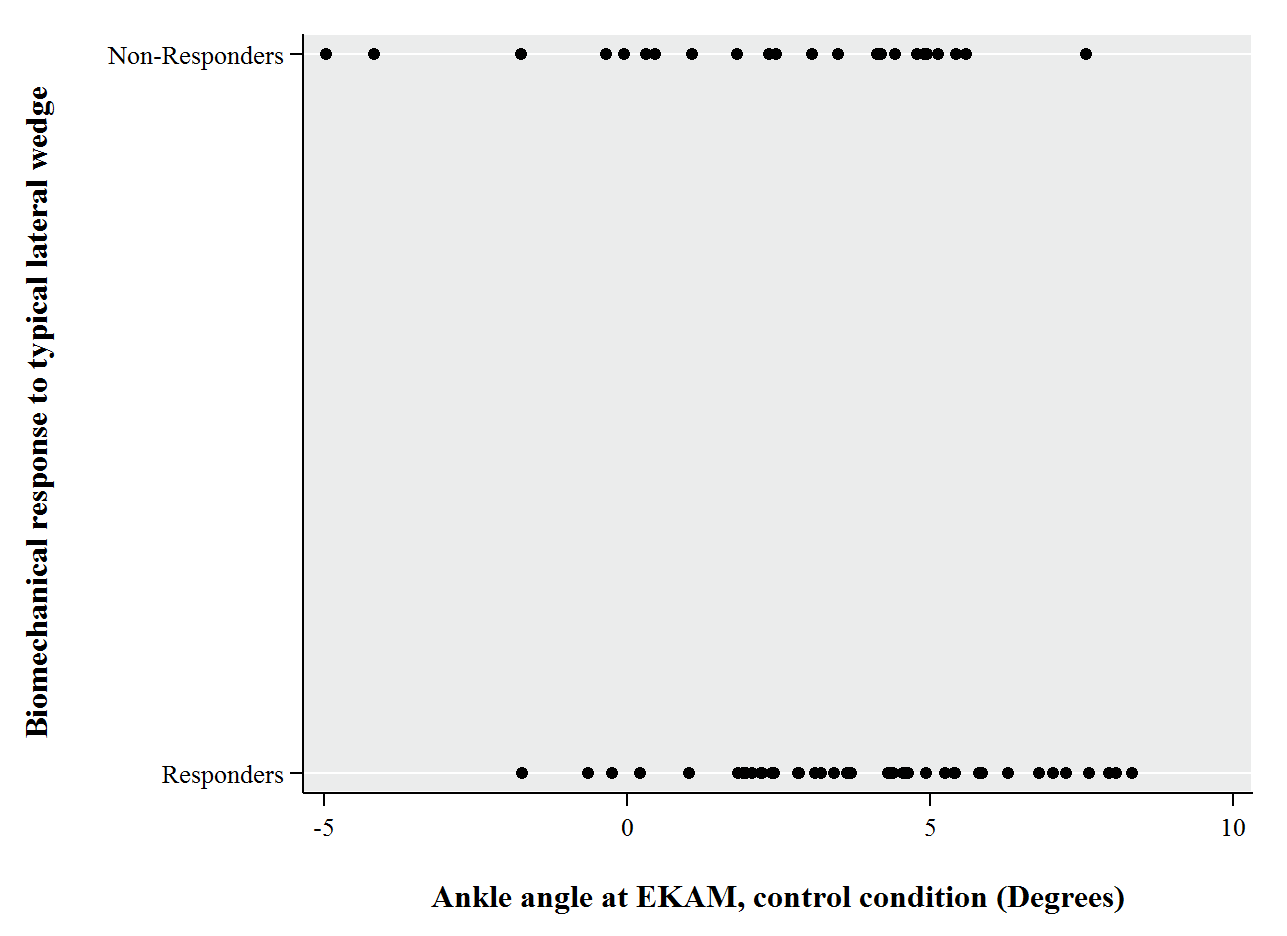

Supplement: Supplementary file 1 [file mmc1.docx]
